# A Machine Learning Approach to Predict Radiation Effects in Microelectronic Components

**DOI:** 10.3390/s24134276

**Published:** 2024-07-01

**Authors:** Fernando Morilla, Jesús Vega, Sebastián Dormido-Canto, Amor Romero-Maestre, José de-Martín-Hernández, Yolanda Morilla, Pedro Martín-Holgado, Manuel Domínguez

**Affiliations:** 1 Departamento de Informática y Automática, UNED, Juan del Rosal 16, 28040 Madrid, Spain; fmorilla@dia.uned.es; 2 Laboratorio Nacional de Fusión, CIEMAT, Complutense 40, 28040 Madrid, Spain; jesus.vega@ciemat.es; 3 Centro Nacional de Aceleradores, Universidad de Sevilla, CSIC, JA, Avda. Tomás A. Edison 7, E-41092 Sevilla, Spain; mrmaestre@us.es (A.R.-M.); ymorilla@us.es (Y.M.); pmartinholgado@us.es (P.M.-H.); 4 Alter Technology TüV Nord, Avda. Tomás A. Edison 4, E-41092 Sevilla, Spain; jose.demartin@altertechnology.com (J.d.-M.-H.); manuel.dominguez@altertechnology.com (M.D.)

**Keywords:** commercial off-the-shelf, degradation prediction, new space, radiation effects, unsupervised clustering

## Abstract

This paper presents an innovative technique, Advanced Predictor of Electrical Parameters, based on machine learning methods to predict the degradation of electronic components under the effects of radiation. The term degradation refers to the way in which electrical parameters of the electronic components vary with the irradiation dose. This method consists of two sequential steps defined as ‘recognition of degradation patterns in the database’ and ‘degradation prediction of new samples without any kind of irradiation’. The technique can be used under two different approaches called ‘pure data driven’ and ‘model based’. In this paper, the use of Advanced Predictor of Electrical Parameters is shown for bipolar transistors, but the methodology is sufficiently general to be applied to any other component.

## 1. Introduction

Electronic devices in space missions suffer different types of damage, including degradation due to the radiation environment. The replacement of components during the mission is not feasible, while some techniques exist to mitigate these effects, in general, success resides in the careful selection of components. That is why one of the most relevant tasks, to qualify and validate the use of devices and materials in hostile radiation environments, are irradiation tests.

Nowadays, commercial companies and governmental space agencies are betting on a new type of market known as “New Space” [1,2]. This new market is depicted, on the one hand, by launching small satellites orbiting in Low Earth Orbit (LEO) and Medium Earth Orbit (MEO). On the other hand, it is characterized by the tendency to use Commercial off-the-shelf (COTS) instead of Radiation-Hardened Assurance devices (RHA) [3]. The difference between COTS and RHA devices lies in the fact that the latest ones have been designed, manufactured, and characterized to work in hostile radiation environments, and also, strategically selected to have homogeneity within the same lot by the manufacturer itself. The use of COTS versus RHA has a clear advantage in terms of cost savings, electrical performance, and lead time. Even so, the standards for space applications [4,5] recommend performing radiation tests to characterize the degradation that the devices will suffer during a mission. This task is not always affordable, so we are immersed in the challenge of finding a new approach. In this paper, the authors present a new technique to predict the degradation of such devices using historical data from previous tests as a complementary alternative to radiation testing.

According to European Cooperation for Space Standardization [4], the three main radiation effects on electronic components are Total Ionizing Dose (TID), Displacement Damage or Non-Ionizing Dose (TNID), and Single Event Effects (SEE). For the purpose of this work, attention will be focused on TID degradation on discrete components. In order to simplify the complexity of the problem, we have chosen to utilize a generic component, such as the bipolar transistor 2N2222 [6], which serves as a representative choice amongst various alternatives. This approach is being applied across numerous upcoming ESA space missions, thereby demonstrating its relevance and applicability in similar contexts.

Specifically, to analyse the damage of samples (microelectronic components) due to ionizing radiation, TID tests are performed in conformance to ESCC 22900 (European Space Components Coordination group) [7], MIL-STD 883 method 1019 [8], or MIL-STD-750 method 1019 [9] on a significant number of samples from the same production lot. These tests consist of measuring different electrical parameters of these samples before and during irradiation test up to the maximum limit established by the mission conditions. For the demonstration proposed in this article, we will refer to the absorbed dose in terms of rad due to the fact that the radiation effect community in electronics tends to use rad instead of Gy, the units of the International System. The absorbed dose depends not only on the incident radiation, but also on the absorbing material (Si, SiO_2_, GaAs…), so the authors define the unit rad(Si) for this work, since the majority of the data included in the database are related to silicon and its use is internationally accepted [10].

Following the recommendations of the European Space Components Coordination group and the US Department of Defence [7,8,9], the process of testing electronic devices on a lot-to-lot basis involves starting each evaluation process from scratch, i.e., on pristine samples, thus not considering previous information on the same type of device. To significantly circumvent this requirement, historical data from previous tests must be used. The immediate effect of this measure could be a temporary reduction in the number of radiation tests, since lot-to-lot testing would apparently be no longer necessary. However, due to the characteristics of the technique developed in this work, APEP, irradiation tests are and would still be necessary.

The use of historical data as a starting point in the design stage is accepted by the community, but it is true that these data usually are not easily found, accessed, or interpreted. It is quite common that these historical data are not available in central publicly accessible databases and/or the databases are mostly out-of-date and/or the data are focused on one organization or manufacturer. To try to mitigate these problems, the ‘Centro Nacional de Aceleradores (CNA)’, a Spanish ICTS (Unique Science and Technology Infrastructures), together with the company ‘ALTER Technology TÜV Nord’ have developed a project to build a specific database: PRECEDER [6]. This is based on the previous work initiated in the frame of the project with the same name (PRECEDER project) [11]. The database is built from the experimental data contained in thousands of radiation test reports. The main goal of PRECEDER is to share the knowledge obtained through data analysis while the Intellectual Property (IP) is kept secure from and forbidden to third parties. Only anonymized data are used for analysis and there are two access levels. On the one hand, free distribution to the already public test reports data will be available on the internet. On the other hand, the creation of a restricted radiation community will be made up of groups that contribute their knowledge to develop a bigger database.

This work makes use of the PRECEDER database in its present form to predict the degradation of electronic components due to radiation by means of machine learning methods. The aim is to make degradation predictions by measuring the electrical parameters of the samples without irradiating the components. This paper shows a particular method to tackle this: the Advanced Predictor of Electrical Parameters (APEP). As a result, four important challenges are attained: destructive tests can be maintained at a minimum, test set-ups are simpler, high-cost reduction of tests is achieved, and high reliability of predictions is possible.

In this work, as a proof of concept for APEP, the bipolar transistor family is used to predict device degradation by simply measuring the electrical parameters without irradiating the samples. Although the analysis presented in this work is based on the 2N2222 device, the authors have performed studies applying different regression models on electrical parameters of other types of devices such as operational amplifiers [12] and optocouplers [13].

Section 2 explains the way in which the radiation tests are performed and gives a short introduction to the PRECEDER database. Section 3 summarizes the objectives of APEP. In addition, some machine learning concepts that are used for the purpose of this work together with two data models for degradation prediction are presented. Section 4 describes in depth the innovative APEP method. Section 5 shows the results of applying the new method to datasets obtained from the PRECEDER database. Finally, Section 6 finishes the study with a discussion about the new technique and its extrapolation capabilities to several types of electronic devices.

## 2. Radiation Tests and the PRECEDER Database

This section introduces the concept of degradation and describes how radiation tests are carried out. Also, the need for creating repositories of radiation tests is discussed. A short description of a particular repository (the PRECEDER database) is presented. As mentioned above, the use of historical data is accepted by the community. However, until now these data have only been used for reference purposes. The great advantage of PRECEDER, as database, is that it is not only a consulting repository, but its data structure also allows all the information to be used for statistical studies, pattern recognition, and machine learning algorithms.

To estimate the degradation of samples, electrical tests are performed. These tests measure certain electrical parameters when the sample is under the influence of radiation. However, it is important to note that normally there could be two types of tests conditions that differ in the biasing state of the sample. On the one hand, irradiation tests can be carried out with the sample properly biased around its operating point. For the purpose of this work, these cases will be referred to as ‘biased samples’. On the other hand, samples can be irradiated with all their pins grounded. Here, these cases will be referred to as ‘unbiased samples’.

Table 1 is an example of some electrical quantities (first column) that can be measured during the tests. In the present case, the objective of these measures is to analyse the changes in the electrical parameters because of the radiation. Each manufacturer determines the working conditions of a sample (column ‘Conditions’ in Table 1) and the specified range of each parameter for the given conditions (columns ‘Min limit’ and ‘Max limit’ in Table 1). It should be noted that in some cases, the manufacturer does not give a specific range but either a minimum or a maximum threshold. Table 1 shows an example proposed by one of the manufacturers that are taken into account for the APEP methodology, such as Microsemi, STMicroelectronics, Analog Devices, Microchip, or Semicoa, among others.

**Figure 1:**
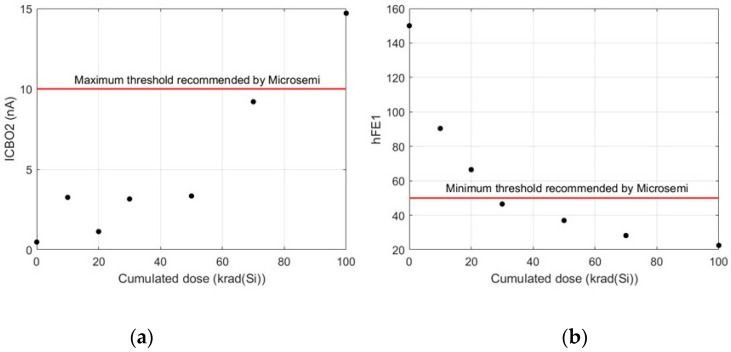
Example of degradation of bipolar transistors 2N2222 by measuring (**a**) the ICBO2 and (**b**) hFE1 parameters at different irradiation doses. It should be noted that degradation does not necessarily mean a decreasing behaviour of the electrical parameters. The dots represent the cumulative dose value.

In this work, the term degradation refers to the way in which one electrical parameter varies with the cumulated dose. Figure 1 shows the evolution of two parameters corresponding to transistors of type 2N2222. The top plot (Figure 1a) represents the evolution of the ICBO2 parameter of Table 1 after six irradiation step doses (10, 10, 10, 20, 20, and 30 krad(Si), respectively, which represent cumulated doses of 10, 20, 30, 50, 70, and 100 krad(Si), respectively). The electrical tests were performed with a VCB = 60 V. According to the recommendations of the manufacturer, the transistor remains in the guaranteed range of the ICBO2 parameter except in the case of 100 krad(Si), where a potential degraded behaviour is identified (ICBO2 > 10 nA). Technically, this means that transistors from the same assembly lot could be used up to cumulated irradiation doses of 70 krad(Si). Their use with cumulated doses above 70 krad(Si) could be possible, but optimal operation is not ensured.

Figure 1b plots the variation in the hFE1 parameter (Table 1) with the cumulated irradiation rate. In this case, the electrical tests were carried out with IC = 0.1 mA and VCE = 10 V. The sample is outside the optimal range with a cumulated dose of 30 krad(Si) (hFE1 < 50). As in the previous example, transistors with the same origin can be used with high confidence up to cumulated irradiation doses of 20 krad(Si). At higher cumulated doses, an optimal behaviour is not guaranteed.

In the examples of Figure 1, both parameters show a degraded behaviour of the transistors. However, when the parameters remain in the specified range, no degradation is recognized.

Typically, to perform radiation tests (with both unbiased and biased samples), parameters are firstly measured without irradiation and, secondly, the levels of radiation are increased to achieve the maximum accumulated dose that is foreseen for the specific test. This is accomplished in a cyclic way where each cycle is made up of irradiation exposure (for example 0 krad(Si), 5 krad(Si), 20 krad(Si), …) followed by an electrical test. It is important to note that exposure to irradiation should be resumed immediately after each electrical test with a maximum delay of two hours between irradiation cycles. In addition, electrical tests must be started within 1 h of stopping the exposure [7].

At the completion of the radiation test, a report is written with relevant details of the test such as traceability data, manufacturing information, number of samples, date of the test, radiation source and variation of the electrical parameters at different irradiation doses. Based on the authors’ experience, it is quite common that the electrical parameters are measured around five different irradiation doses. However, the number of electrical parameters and the number of doses strongly depend on the type of mission for which the specific samples will be used.

The test reports can be collected into repositories that can provide fundamental support as user guides for designers and engineers of different projects. The PRECEDER database represents an easy form to access and interpret the available data on previous radiation test results. PRECEDER is not only a repository of documents but also the first European database with parameterized and classified data for statistical analysis. The information available in the database comes from a wide variety of documents (test reports, papers, slides, etc.), and therefore, it was necessary to treat them to ensure the uniformity and consistency of the information in a unique data structure. PRECEDER’s analysts have digitalized each individual report by properly labelling and categorizing the data. These are not limited to electrical measurement results, they also include data relevant to the test, such as polarization conditions, radiation test conditions (dose rate, source type, etc.), and, when possible, traceability information (manufacturer, date code, diffusion lot, etc.), as this information acts as a high-level filter for better data screening. The PRECEDER database is continuously updated from whatever available source. Nowadays, this database consists of thousands of parameterized and classified documents.

## 3. APEP Objectives and Data Models

As was mentioned in the introduction, this work describes a novel and innovative method (APEP) to predict the degradation of electronic components due to radiation. The aim is to make predictions without the usual restriction of using components from the same lot. In this way, all past existing information concerning a specific type of electronic component can be used in the prediction process regardless of assembly lots and diffusion/wafer.

After the method description, the proof of concept is carried out with bipolar transistors of the type 2N2222. Although the tests are applied to this type of device, it should be emphasized that the technique is general enough to be applied to other electronic components (diodes, MOSFETs, optocouplers, operational amplifiers, PWMs…), since work using regression models and statistical analysis in both bipolar transistors [6] and other types of devices [12,13] has yielded satisfactory results.

Given an electronic component, the aim of APEP is to forecast the values of their electrical parameters, (as the ones shown in Table 1), at any radiation dose in order to infer its degradation with the irradiation. To this end, the main objective is to use a single input for the degradation prediction of each parameter, instead of measuring the amplitudes at several doses. The strong proposal of APEP resides in considering that this single input can be the electrical parameter value that is measured in the absence of irradiation. Consequently, the samples do not need to be irradiated at all and, therefore, the experimental set-up to determine degradation is quite simple in comparison to modern requirements.

Predicting degradation without sample irradiation is an important challenge. To accomplish such an objective, machine learning techniques are useful and, in particular, automatic classification methods are used. Therefore, it is important to review some machine learning concepts related to the method described in this study.

To tackle automatic classification problems [14,15,16], a pair (x_i_, y_i_) represents each observation (or pattern), where x_i_ ∈ R^m^ is the feature vector and y_i_ is the label. The feature vector is the set of features that characterize the pattern, i.e., attributes managed by computers that are of distinctive nature. The label denotes the class to which the pattern belongs to, i.e., y_i_ ∈ {C_1_, C_2_, …, C_3_} where C_j_, j = 1…L are the existing classes.

**Figure 2:**
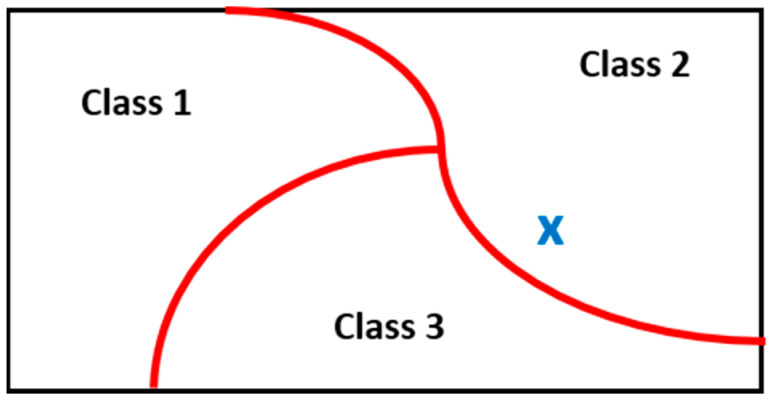
Conceptual example of a supervised classification system in a two-dimensional feature space and three classes. The training process estimates the separation frontiers and new observations are classified according to its location in the feature space. In this case, the blue cross pattern is classified as class 2.

Any automatic classification system requires a training process (where the more observations the better) to obtain the so-called model. In the case of supervised classifiers [17,18,19], the labels of the training observations are known. The training process allows for determining the separation frontiers in the feature space (R^m^) that delimit the borders between classes (Figure 2). After the training, any new observation (x,y) (blue cross in Figure 2), with known feature vector x but unknown label y, is used as input to the model to estimate the label. The feature vector is mapped into the feature space and the observation is assigned to one and only one class depending on the relative position to the frontiers (Figure 2).

In the case of unsupervised classifiers (also known as clustering) [20,21,22,23], given a set of observations (x_i_, y_i_), all feature vectors x_i_ ∈ R^m^ are known but the labels only are known at the end of the training process. The training process groups the observations into clusters (or classes) according to some criterion. In other words, the patterns are grouped into sets based on the properties derived from the clustering criterion, which discriminatively allows for distinguishing one set from another.

The APEP method to predict the degradation of samples, (i.e., to forecast the electrical parameters at several doses), is split into two sequential steps: clustering and sample classification. The clustering step requires a well-known training dataset made up of irradiated samples to perform an unsupervised classification system. This classifier reveals the number of different clusters (or groups) that can be recognized in the training dataset. Each cluster is made up of the samples most likely to be similar in terms of degradation and each cluster is represented by an average model of degradation. These average models allow for recognition of the several degradation patterns that are present in the training dataset.

After finishing the clustering step, any new sample can be assigned to one and only one cluster and the average degradation of the cluster is assumed to be the one of the new sample. To assign a sample to a cluster, the concept of nearest-neighbour is applied. Given a sample to predict its degradation, the new sample is supposed to belong to the cluster of its nearest-neighbour sample (in the terms described in Section 4.4) in the training dataset.

As can be seen from the method description, the training samples are essential elements in APEP. The training samples have to be represented by feature vectors to perform the clustering step. But, the first question is, which are the relevant features? The answer to this can be found in the reports corresponding to previous radiation tests and contained in the PRECEDER database. These reports store all the details of past electrical tests. For the purpose of APEP, the relevant data to take into account are the several electrical parameter values at their corresponding irradiation doses.

Once the relevant features are defined, the next decision that requires APEP is to designate the feature vectors. For the sake of simplicity, the feature vector components are assumed to be the electrical parameter amplitudes corresponding to specific irradiation doses. In this way, by defining the reference doses, the dimension of the feature space is determined. For instance, if the reference doses are 0 krad, 10 krad, and 100 krad, the resultant feature space has a dimension of 3. In the case of 0 krad, 5 krad, 10 krad, 20 krad, 50 krad, and 100 krad, the dimension is 6 and so on.

This definition of feature vectors allows for the development of pure data-driven approaches to perform the clustering step. However, it implies that all samples must have measurements of the electrical parameters for all the required doses. Due to the heterogeneity of the radiation tests, the requirement of data at specific doses can be an issue. There can be a lot of tests with electrical parameters at disjoined doses and, therefore, the number of available samples at particular doses can be insufficient to get reliable clustering models.

An alternative to pure data-driven models is to use model-based approaches. This signifies that all electrical parameter measurements of a sample can be fitted to an explicit model. This is important because samples can have electrical tests at very different irradiation doses and none are discarded due to a lack of data at specific doses. The only condition required to fit the experimental data is the minimum number of measurements to be able to fit the specific model with a certain level of confidence. In this way, the feature vector that represents each sample is made up of the coefficients of the respective models, thereby ensuring the same dimensionality for all samples. Table 2 shows some models that can be used to represent the evolution of electrical parameters with the irradiation dose.

## 4. APEP Method Description

As was mentioned previously, the objective of this work is to show a particular method to predict the degradation of samples without accomplishing the irradiation process. By using machine learning methods, the prediction of sample degradation is possible. For this purpose, a database of samples with well-known degradation measurements is needed. Even though the selected data are from 2N2222 devices, they come from a variety of sources, due to this, they may not share the same manufacturer, the same assembly lot, or the same wafer. Because the nature of these data is so general, i.e., they do not have the same origin, it is possible to distinguish different patterns that allow finding higher-level relationships. The relevant information for the objective of this study is to know the values of electrical parameters at different doses, as is shown in Figure 1 for the ICBO2 and hFE1 parameters, respectively.

This section is split into five subsections. The first one (Section 4.1) describes the sample database that has been used to test the APEP method. On the other hand, as established in Section 3, the global prediction method consists of two sequential phases: clustering of known samples and forecasting the amplitudes of electrical parameters at several doses when a new sample is given. Section 4.2 gives an example of clustering and its interpretation. Section 4.3 details the unsupervised clustering process and how an optimal number of clusters is chosen. Section 4.4 defines the several steps of the APEP prediction process. It is important to note that all examples given in Section 4.2, Section 4.3 and Section 4.4 refer to a data-driven approach. A model-based approach for APEP is discussed in Section 4.5.

### 4.1. Use Cases to Test the APEP Method

The database used in this study is the PRECEDER database, which has been described in Section 2. In particular, bipolar transistors of the type 2N2222 have been chosen for the APEP proof of concept. The selection includes all data stored in the database from different sources for the 2N2222, where the data have been filtered and separated according to the polarization condition during radiation exposure: biased and unbiased samples. The method has been applied to four different electrical parameters of the 2N2222 devices: hFE1, hFE2, hFE3 and hFE4 (see Table 1). Therefore, in this work, the APEP method has been tested for eight different use cases: four parameters and two irradiation conditions (biased and unbiased samples).

Owing to the fact that the database can contain several records (i.e., measurements for several electrical parameters at different doses) from the same sample (bipolar transistor), henceforth, the measurements of electrical parameters at different doses will be referred to as ‘database records’ or simply ‘records’.

It is important to note that, in general, the PRECEDER database does not include simultaneous measurements of the four electrical parameters and irradiation conditions for all the samples. Therefore, each use case can have different number of records in the available database. On the other hand, to ensure good data quality, a data cleaning process is an important step. In particular, records that show outliers in their electrical amplitudes have been filtered. Additionally, other test conditions which potentially could affect the degradation, such as the biasing conditions and the dose rate, are considered before selecting the samples for the training process. This has been carried out case by case and Table 3 shows the resulting datasets of records for each use case. Also, it is important to remark that the records for a given electrical parameter and a given irradiation condition can have data for different irradiation doses between them.

As usual to validate the models derived from machine learning approaches, the records that belong to each use case are split into two datasets. The first dataset is the training dataset that, in the APEP case, allows performing the unsupervised clustering process (i.e., to obtain the model) as a previous step to forecast the amplitude of specific electrical parameters and irradiation condition of new samples. The second dataset is used as the test set to validate the model generated in the training phase. The records of a test set do not intervene in the clustering process (i.e., in the training phase) but their degradation is known. Therefore, they are used to test the prediction method and to obtain success rates.

### 4.2. Example of Clustering and Interpretation

**Figure 3:**
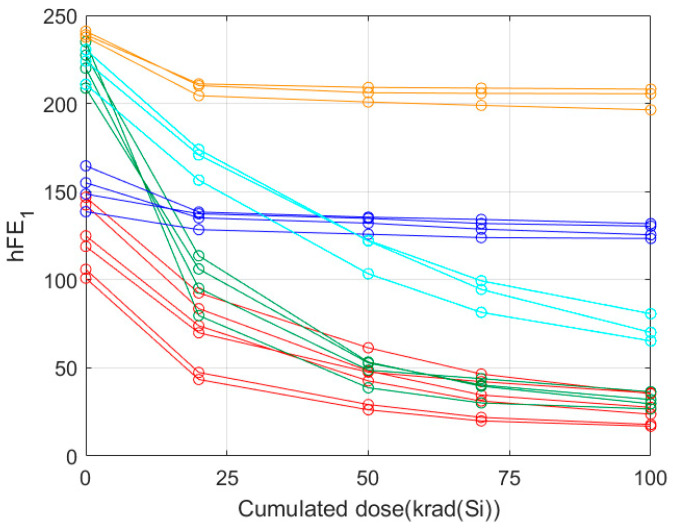
Example of 20 samples that show the presence of five different classes, each one represented by a specific colour: orange, cyan, green, blue, and red.

In each use case, the unsupervised clustering process groups the training records into several classes that are characterised by a similar degradation pattern to the electrical parameter. To illustrate the exercise of the unsupervised clustering, we selected 20 training records of the total available corresponding to the parameter hFE_1_ in biased conditions (Table 3). Figure 3 is illustrating how these 20 training records, represented by their respective feature vectors, have been grouped into five clusters using an unsupervised clustering process. The clustering provides us with the possibility to define the average behaviour that we used then to establish a prediction interval for each dose with a specific confidence of level. The calculation of the average behaviour for each individual cluster defines the behaviour at each irradiation dose as the mean value of the amplitudes at that dose.

**Figure 4:**
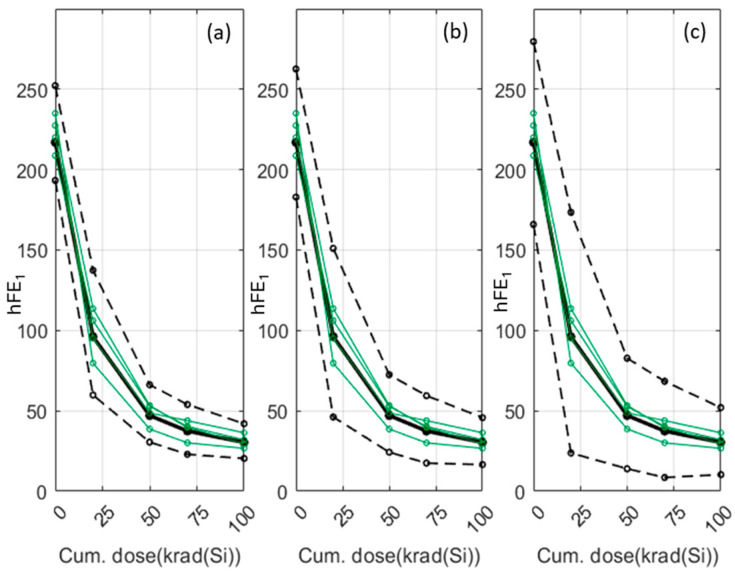
Average degradation of the green cluster of Figure 3 with three different confidence levels: (**a**) 0.9 (**b**) 0.95 (**c**) 0.98.

To show the use of the group mean behaviour, we use the cluster formed by the set of green records shown in Figure 3. The result is shown in the Figure 4, where the group mean behaviour is represented by the black solid line. For this example, three intervals have been calculated from three different confidence levels (0.9, 0.95, 0.98) and the area between the dashed lines, where the width of the interval increases with the confidence level.

At this point, it is important to give an interpretation of the clustering process. Figure 3 plots the degradation of 20 different samples corresponding to the hEF_1_ electrical parameter. The unsupervised classification groups together the samples whose degradation shows a high similarity, where the term ‘similarity’ refers to the similar evolution of the electrical parameter with the irradiation doses. The similarity of the degradation can be quantified by means of the normalised inner product [24,25]:

(1) $$S_{uv}=\left|\cos\alpha \right|=\frac{\left|u_{D}\cdot v_{D}\right|}{\left\|u_{D}\right\|\cdot \left\|v_{D}\right\|},\;0\leq S_{uv}\leq 1$$

where $u_{D}$ and $v_{D}$ are, respectively, the feature vectors that show the degradation of an electrical parameter corresponding to two different samples. The case $S_{uv}=1$ means that the degradation of both feature vectors is exactly the same. As the feature vectors differ, the similarity decreases. Table 4 shows the average similarities among the samples of Figure 3 within the same cluster (column 2) and between the samples of different clusters (column 3).

Therefore, the main outcome of the clustering process is to provide the several degradation patterns that are present in the training records. In the example of Figure 3, five different degradation patterns are identified.

### 4.3. APEP First Phase: Unsupervised Clustering Process and Determination of the Optimal Number of Classes

This section presents the first phase of the APEP method: the clustering of the training samples to determine how many degradation patterns appear in the data.

First of all, it should be mentioned that not all the records of a specific electrical parameter and particular biasing condition have the same number of measures. For instance, in Table 3, there are 223 records of the hFE_1_ parameter corresponding to biased samples. To carry out the unsupervised clustering, the feature vectors not only must have the same dimension, but also their components have to represent the same irradiation doses. However, we should keep in mind that not all records can have data on the same accumulated dose number. Moreover, even if there are records with the same number of measurements, the value of accumulated doses may not coincide. To solve this problem, a normalisation algorithm that allows clustering of cumulative dose values within a 10% deviation, which working conditions are allowed by the [8,9] standards, is applied. 

Given a training dataset of records (with the same dimensionality and corresponding to the same irradiation doses), the unsupervised classification of such records provides the grouping of the training records into a number of clusters, as shown in Figure 3. In particular, the clustering algorithm used in this paper has been a hierarchical clustering algorithm [26]. However, it is important to note the need to find a good enough partitioning of the training dataset. In other words, the initial data (with any clustering algorithm) can be split in 2, 3, 4 or more classes but it is necessary to some kind of criterion to get an estimation of the quality of the clustering results and to evaluate the ‘*correct*’ number of groups in the data.

There are several methods to address this, for instance, Rand index [27], cophenetic correlation coefficient [28], upper tail rule [29], silhouette plot [30], gap statistic [31], and S-Dbw validity index [32]. The latter has been used in this study. The advantage of the S-Dbw index against others resides in the fact that the S-Dbw index not only evaluates how compact and separated are the clusters but also it considers the density of the clusters. In addition to this, the S-Dbw index is independent of the clustering algorithm used to split the data.

To select a good enough number of clusters with the S-Dbw index, this index is computed for each data partition between 2 and *K* clusters. Therefore, *K*–1 validity indexes are obtained and, as a general criterion, the data partition corresponding to the minimum index gives the optimal number of clusters.

**Figure 5:**
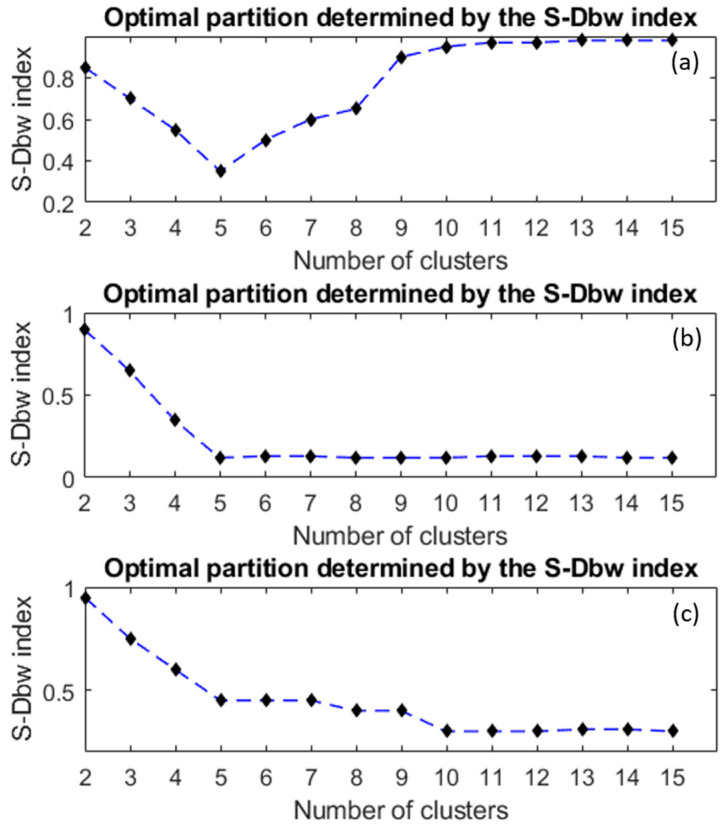
Examples of the selection of a good enough number of clusters.

Figure 5a shows an example of clustering whose optimal partition, as determined by the S-Dbw index, is to split the data into five classes. On the other hand, Figure 5b corresponds to a case in which there is not a clear minimum. In these cases, the number of clusters to consider is where an ‘elbow’ in the curve appears or the place where the curve becomes practically flat. In the example shown in Figure 5b, the optimal number of clusters is also five. Finally, the case plotted in Figure 5c shows that the ‘elbow’ in the curve means that five clusters are a realistic number of clusters. However, other ‘elbows’ at eight and ten also might provide interesting clusters.

### 4.4. APEP Second Phase: Steps to Predict the Degradation of New Samples

To carry out the second phase of APEP, it should be reminded that the main objective of this method is to be able to make predictions without irradiating the new samples. According to this, the prediction process can be summarised as a four-step process (for both biased and unbiased samples):

1. Measuring the electrical parameter amplitude of the new sample without applying irradiation: A(0).
2. Determining the record of the training dataset (reference record) whose amplitude at irradiation 0 is the closest one (nearest-neighbour) to the amplitude obtained in step 1.
3. The degradation of the electrical parameter of the new sample is assumed to be the one corresponding to the reference record, where the degradation of the reference record is the average degradation of the cluster to which the reference record belongs to. The amplitude of the average degradation at dose 0 krad is represented by AD(0).
4. The electrical parameter at the different doses corresponds to one of the average degradations of step 3 but all the amplitudes are displaced with an offset of A(0)–AD(0).

**Figure 6:**
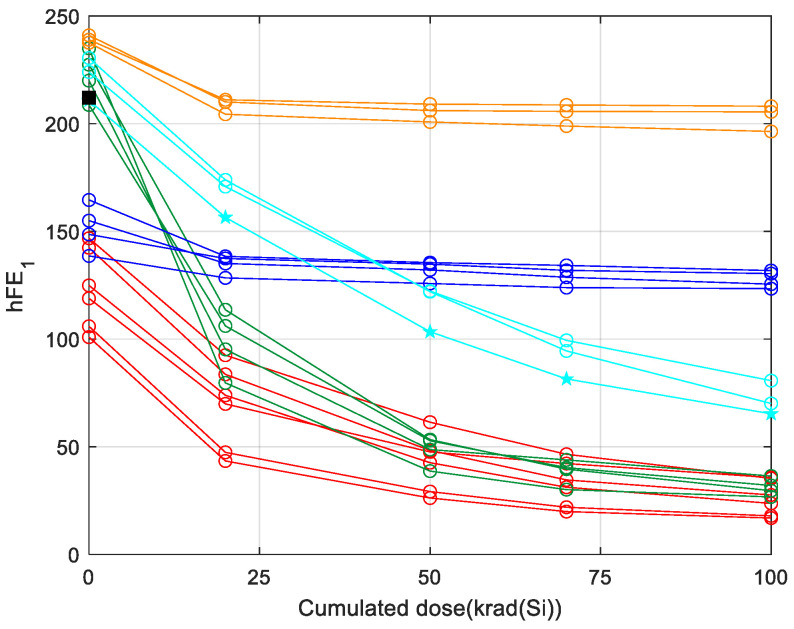
Example to predict the degradation curve of a new sample. The only requirement is to measure the electrical parameter amplitude without irradiating the sample (black square).

Figure 6 shows an example of the prediction method of a new sample according to the above four steps. In the example, there are 20 samples whose feature vectors have a dimension of 5. The amplitudes of the hFE1 parameter are given at five accumulated doses: 0, 20, 50, 70, and 100 krad. The clustering of the training samples is the one shown in Figure 3. The electrical parameter of the new sample is measured without irradiation (step 1) and the obtained amplitude, A(0), is represented by the black square in Figure 6. The second step determines that the nearest-neighbour amplitude at 0 krad corresponds to a training sample whose markers are pentagrams (this is the reference record). The reference record belongs to the cyan cluster and, therefore, the degradation of the new sample is assumed to be the average degradation of the cyan cluster.

**Figure 7:**
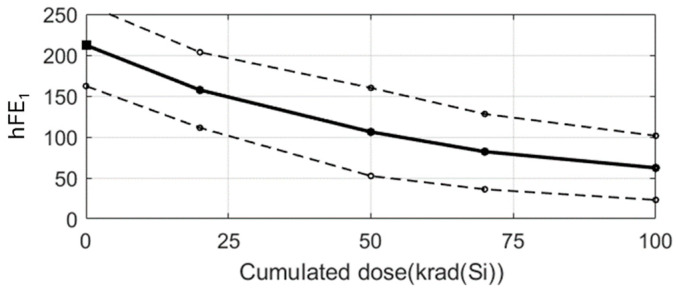
Predicted degradation curve corresponding to a new sample whose amplitude at 0 krad is shown in Figure 6. The plain line is the average degradation of the cyan cluster after a displacement of A(0)–AD(0). The dashed lines determine the prediction interval of the cyan cluster.

Figure 7 shows the predicted degradation of the new sample. It is important to note that the degradation curve is estimated as the average degradation of the cyan cluster but is translated to start in the amplitude that has been measured with no irradiation. In other words, as stated in step 4 above, the average degradation of the cyan cluster is moved by an offset given by A(0)–AD(0) where A(0) is the amplitude that has been measured without irradiation and AD(0) is the amplitude of the reference record at an irradiation dose of 0 krad. As can be seen, the rest of the measurements taken at different cumulative dose levels fall within the confidence interval calculated for the cluster in which they were classified.

### 4.5. APEP Model-Based Approach

So far, all examples in this section are based on a pure data-driven approach. However, a model-based approach is more general and flexible in the sense that the database of records can be completely heterogeneous. This heterogeneity is related to the fact that the records (i.e., the amplitudes at several doses) can be of different dimensionalities and the measurements can correspond to very different irradiation doses. Now, it is not necessary to require that all records in the same cluster have the same dimension. The only restriction to use a record in a model-based approach is to have the minimum number of points to be able to fit the experimental data to a specific model. For example, for the exponential model, the minimum number of data points is three, but for the sigmoid model, four points are required to fit the model. From a conceptual point of view, the prediction process is exactly the same as what has been described for the data-driven case.

**Figure 8:**
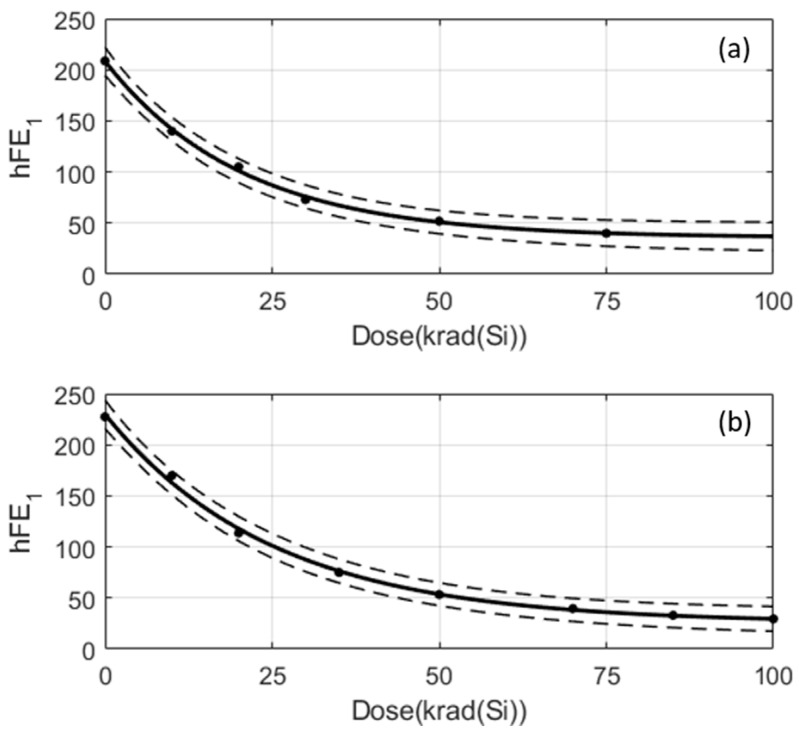
(**a**) Six experimental points are fitted to an exponential model. (**b**) Eight experimental points are fitted to an exponential model.

Figure 8 shows two examples of degradation that follow an exponential model (Table 2). Two different records for hFE_1_ biased (Table 3) with different dimensionality are taken for this example. In both cases, the confidence level of the fit is 0.95. The respective coefficients, after fitting the model, are $a_{1}=208.32$, $a_{2}=35.72$ and $a_{3}=0.05$ for the top plot and $a_{1}=229.92$, $a_{2}=25.78$ and $a_{3}=0.04$ for the bottom plot. Although both examples show a different number of experimental points, by approaching the data to an exponential model, both examples are represented with only three dimensions and the respective feature vectors are $\left(208.32,35.72,0.05\right)$ and $\left(229.92,25.78,0.04\right)$.

**Figure 9:**
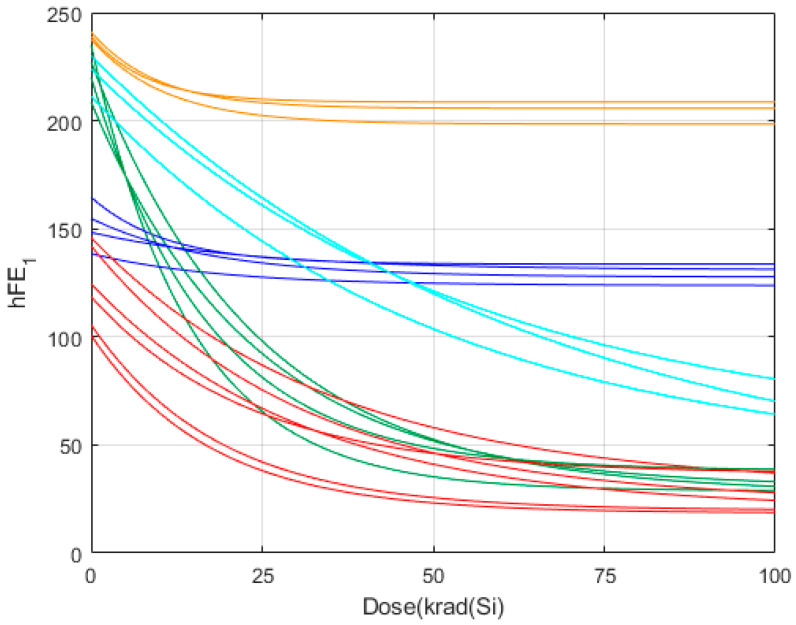
Example of 20 samples fitted to an exponential model that show the presence of 5 different degradation patterns as in Figure 3. The samples that form each cluster are the same as in the data-driven approach.

In order to compare the two approaches, we take the same 20 training datasets that were selected in Section 4.2 as an example of clustering. First it is necessary to fit each record with a model, in this case exponential degradation. Then, the same unsupervised clustering in Figure 3 is applied. Figure 9 shows the clustering procedure using the model approach. The difference is that all the data have been fitted now to an exponential model and, therefore, the dimension of the feature vectors in this case is 3. It is important to note that in the data-driven approach of Figure 3, the unsupervised learning process groups together exactly the same training samples.

**Figure 10:**
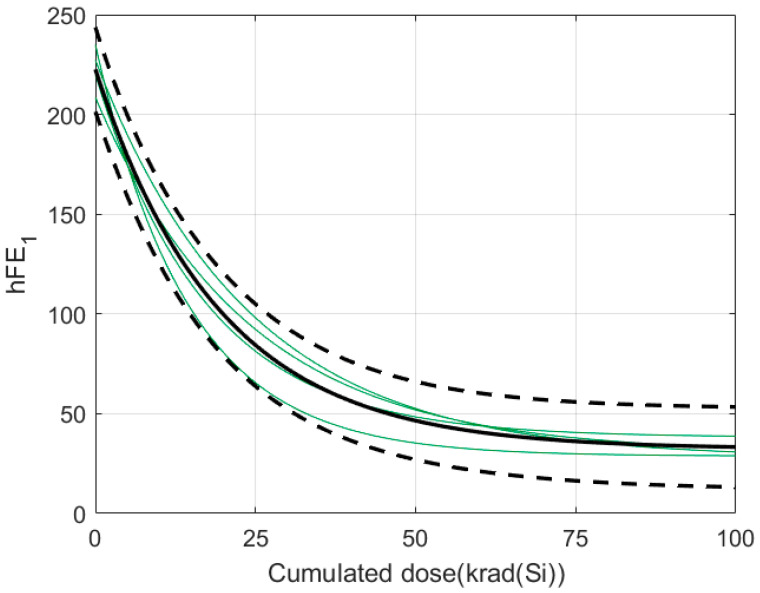
Average degradation pattern in a model-based approach with 0.95 confidence level.

As was done in the data-driven approach, we will calculate the average degradation of the cluster. Figure 10 shows the average degradation pattern of the green cluster of Figure 9 with a 0.95 confidence level. The four records that made up the cluster are represented, respectively, by the green lines. The average pattern is the plain black line, and the dashed black bold lines are the confidence level limits. As it can be seen, the completed cluster is within the confidence interval.

**Figure 11:**
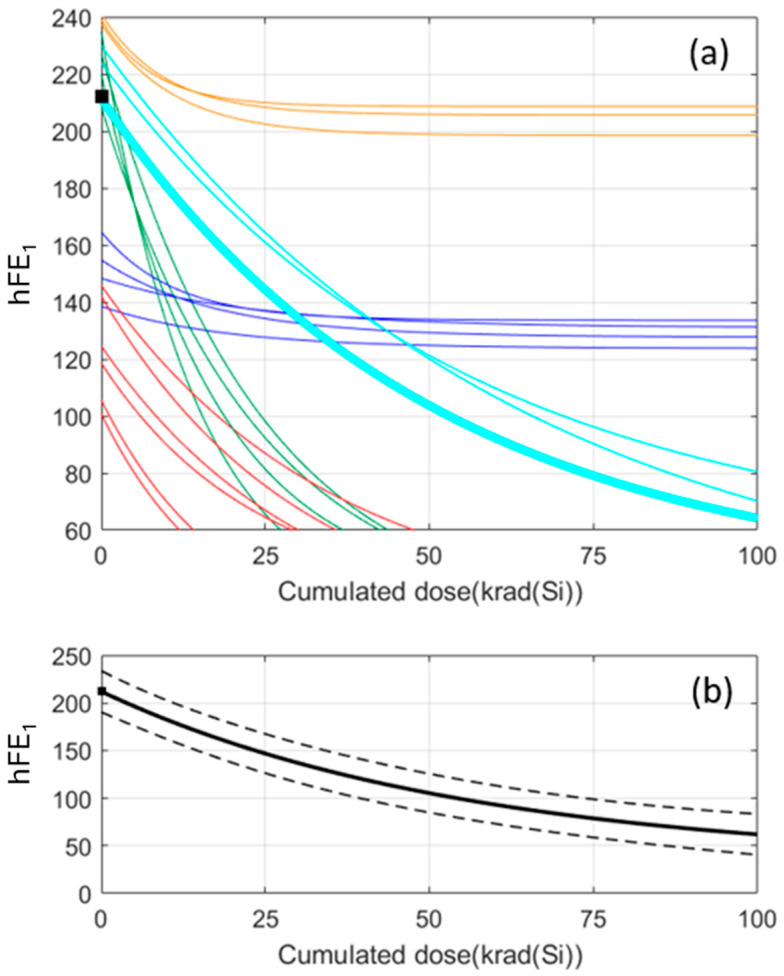
(**a**) The hFE1 parameter of a new sample at 0 krad has been measured and it is represented by the black square. (**b**) Prediction of the hFE_1_ amplitudes by means of the average degradation pattern of the cyan cluster. The dashed lines are the prediction interval.

Analogous to what was done previously, we take the same validation data record used in the data-driven approach. The results are given in Figure 11. The black square in Figure 11a is the hFE_1_ amplitude at 0 krad of a new sample whose degradation is to be predicted. Its reference record (nearest neighbour) is plotted with a thicker cyan line and its degradation pattern is the one in the cyan cluster. Figure 11b shows the prediction of the hFE_1_ amplitudes in the range of doses 0–100 krad. As in the data-driven approach, the prediction is obtained by moving the average degradation pattern with an offset given by A(0)–AD(0), where A(0) and AD(0) are, respectively, the amplitude that has been measured without irradiation and the amplitude of the reference record at an irradiation dose of 0 krad. It should be emphasised that the prediction intervals are narrower in the model-based approach. If we compare the results obtained in Figure 11 with those in Figure 6 and Figure 7, we can observe many similarities. On the one hand, the measure has been clustered in the same group, although a different approach is used. On the other hand, the rest of the measures are within the confidence interval calculated using the latter approach, as obtained above.

## 5. Application of APEP to Bipolar Transistors from the PRECEDER Database

This section describes the results of applying the degradation prediction method that has been presented in Section 4. As mentioned, the method has been tested for eight different use cases, corresponding to four electrical parameters (hFE_1_, hFE_2_, hFE_3_, and hFE_4_) and two irradiation conditions (biased and unbiased). Therefore, eight different datasets of records (see Table 3) have been taken into account. Each one of the use cases has been considered under two different scenarios: a pure data-driven approach and model-based approach. In both scenarios, the feature vectors are obtained from the database records to perform the two sequential steps of the prediction method: unsupervised clustering and degradation prediction of new samples.

An essential part of this paper is to assess the prediction capability of APEP. The standard process to validate machine learning models is to split the available data into two subsets: training and test. APEP uses the training set of records to create the unsupervised clustering models. The test set is used to evaluate the success rate of the predictions as the amplitudes at different doses are known.

The percentage of records for training/tests has been 70%/30% in all the cases of use. Therefore, the success rates are computed with 30% of the records available for each use case. However, to avoid any bias in the selection of training/test records, 100 datasets of training/test records have been randomly chosen in each use case. Therefore, as shown later, the success rates in each use case are computed through measures of the central location from 100 different test sets.

An important point to clarify is how to define the term ‘*success rate*’. Due to the fact that the objective is the prediction of electrical parameter amplitudes at several irradiation doses, a natural way to define the success rate can take into account the number of predicted amplitudes that are compatible with real measurements. In this sense, the key term is ‘*compatible*’.

**Figure 12:**
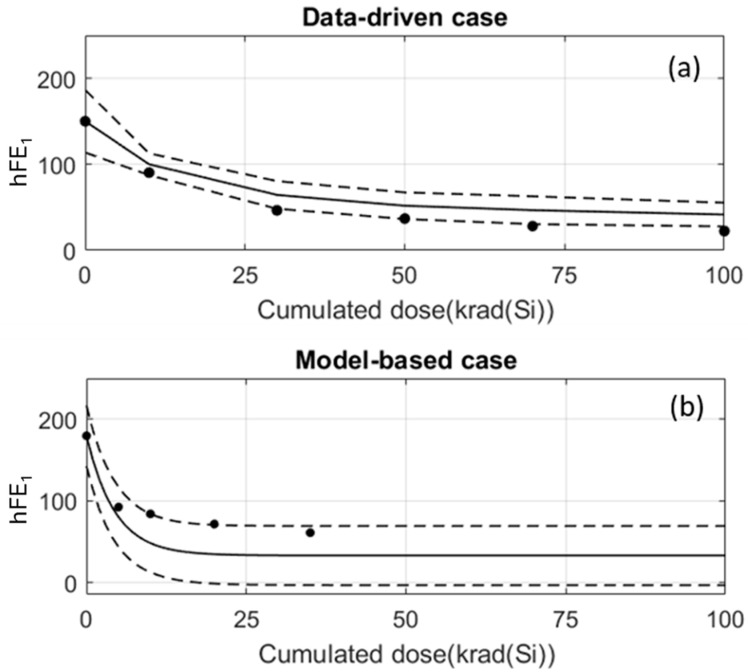
Dashed lines show the limits of the confidence regions of a prediction. The black circles are real measurements of test samples. (**a**) Data-driven case and (**b**) Model-based case.

The real electrical amplitudes of the test records are known, and they will be used to compute the success rate. It should be reminded that given a sample to predict and its electrical amplitude at 0 krad, APEP assigns the average degradation of its reference record as a prediction. In particular, the average degradation is displaced as an offset in such a way that the prediction at 0 krad is just the value of the electrical parameter at 0 krad. Figure 7 and Figure 11b show this. But, it is important to emphasise that the average degradations are computed with the records of the training set and, therefore, regions of confidence can be established (Figure 4 and Figure 10). When a prediction is performed, this region of confidence has to be taken into account to assess how *compatible* the prediction is with real measurements. Figure 12a shows the predicted amplitudes (plain line) and the confidence region (dashed lines) together with the real amplitudes (black circles) of a test sample in a data-driven case. The predictions are evaluated at the doses at which real measurements are available. In the figure, there are measurements at six different cumulated doses, where three of them are inside the confidence region. A prediction within the confidence region is considered to be *compatible* with a real measurement. Therefore, the success rate in this example is defined to be $\left(3/6\right)\cdot 100\%=50\%$. In a similar way, Figure 12b plots a model-based example in which the success rate for a distinct sample is $\left(4/5\right)\cdot 100\%=80\%$.

To take into account all the experimental measurements of a test set, the term ‘*success rate*’ is defined as

(2) $$\mathrm{success}\;\mathrm{rate}\left(\%\right)=\frac{\#\mathrm{measurements}\;\mathrm{inside}\;\mathrm{the}\;\mathrm{confidence}\;\mathrm{regions}}{\#\mathrm{measurements}}\cdot 100$$

For a high success rate, the number of measurements within the confidence interval should be as large as possible. This requires sufficient reliability in the data used for training so that the interval defined from the average degradation of the different clusters is well defined. However, as it was mentioned above, 100 different test sets have been randomly chosen to validate the APEP method. Therefore, 100 different results are available per use case. In particular, four statistical parameters are used to characterize the success rate in each use case from the 100 test sets: minimum value, median, mean, and maximum value.

Section 5.1 and Section 5.2 present, respectively, the APEP results in data-driven and model-based approaches.

### 5.1. Data-Driven Approach

According to Section 3, it is important to remind that the feature vector components are either the electrical parameter amplitudes corresponding to a common set of cumulated irradiation doses (in a data-driven approach) or the coefficients of a specific model (in a model-based approach). The clustering step is a critical phase to discover, in the clearest way, the variety of different classes (or degradation patterns) that are present in the training data. In this respect, the more training records the better.

It should be noted that the lack of enough training data can jeopardize the recognition of an optimal number of clusters. The issue comes from a double fact. On the one hand, features that really belong to the same class can be classified into different clusters. On the other hand, features that really do not belong to the same class are put together in the same cluster.

The data-driven approach requires that all feature vectors derived from the dataset of records have the same dimensionality (number of components). However, as was mentioned in Section 4, the records within a dataset are not homogeneous, i.e., they do not have data corresponding to a common set of irradiation doses. Unfortunately, this means that not all the records can be used for training/test purposes. It is necessary to find the largest set of records with the largest number of common irradiation doses from each dataset. After this filtering process, the largest set of records is obtained in all use cases for six common irradiation doses: 0, 10, 30, 50, 70, and 100 (in krad). Table 5 extends Table 3 to show the remaining records after the filtering of records (column 4). On average, only 29% of the initial records of biased samples are retained for training/test purposes. In the case of unbiased samples, the percentage is about 50%.

**Figure 13:**
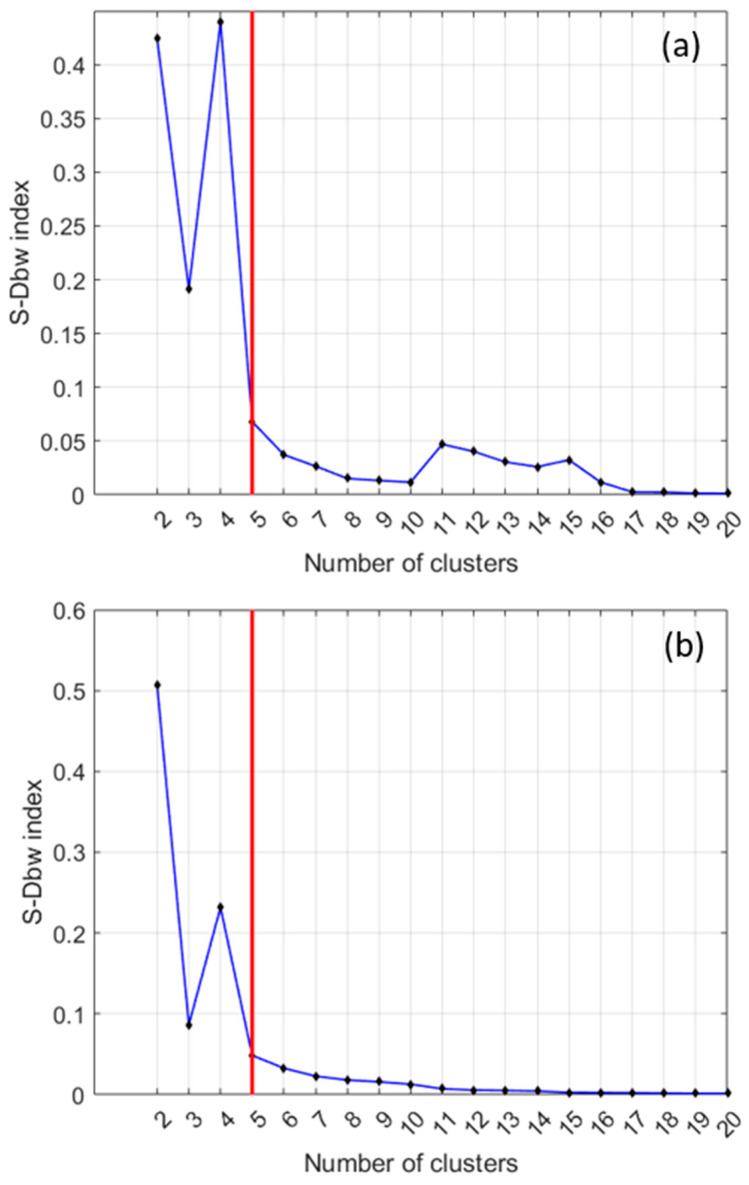
(**a**) S_Dbw index for the case hFE_1_ of biased samples. (**b**) S_Dbw index for the case hFE_4_ of unbiased samples.

Figure 13 shows two examples of the selection of an optimal number of clusters (red line) provided by the S_Dbw index. The examples correspond to the cases hFE_1_ of biased and hFE_4_ of unbiased samples, respectively. In both cases, the optimal partition of the training set is five clusters. It should be mentioned that the partition in five clusters is the typical one that has been obtained in the eight use cases of the data-driven approach.

The number of clusters obtained was used to create models, which were then applied to the test data. Table 6 presents a summary of the success rates of APEP with the data-driven approach.

### 5.2. Model-Based Approach

As explained in the above paragraph and Table 5, the main drawback of a pure data-driven approach is the important reduction in the number of available records to train/test the corresponding models. This is a consequence of having to select the largest number of records with the largest number of common irradiation doses. To overcome this difficulty, a model-based approach can be used. All the use cases in this work are compatible with an exponential degradation model. Consequently, the feature vectors that represent the records have a dimension of 3 and their components are the coefficients $\left(a_{1},a_{2},a_{3}\right)$ of the model (Table 2). Therefore, to fit any record to an exponential degradation model, records with enough numbers of amplitudes can be fitted with a given level of confidence (Figure 8). In this way, the amplitudes of any dose can be estimated (Figure 9). It should be emphasised that in this approach, the several records do not need to have amplitudes at the same irradiation doses.

It is clear that the use of a model-based approach is less restrictive in relation to the availability of records for training/test purposes. In this case, only electrical parameter records with less than the required number of irradiation doses are discarded. Table 5 (column 5) shows the available records for training/tests from the initial set of records. On average, above 71% of the initial biased samples can be used while the percentage is larger than 76% in the case of unbiased samples.

**Figure 14:**
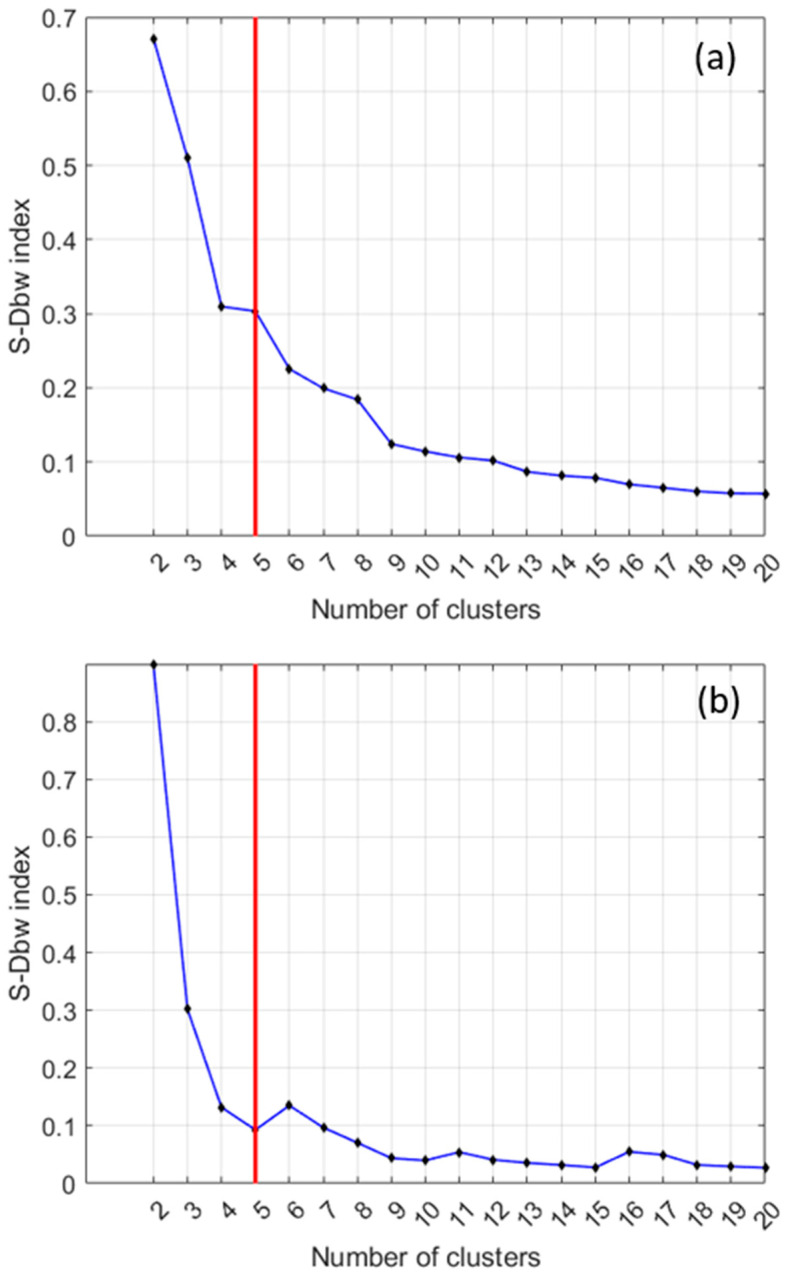
(**a**) S_Dbw index for the case hFE_2_ of biased samples. (**b**) S_Dbw index for the case hFE_3_ of unbiased samples.

Figure 14 shows two examples of the selection of an optimal number of clusters provided by the S_Dbw index in the model-based approach. The plots correspond, respectively, to the cases hFE_2_ of biased and hFE_3_ of unbiased samples. Again, as in the data-driven approach, the typical partition in all cases is five clusters.

The number of clusters obtained was used to create models, which were then applied to the test data. Table 7 presents a summary of the success rates of APEP with the data-driven approach.

## 6. Conclusions

APEP is a novel method that has shown its capability and reliability to predict the degradation of electrical parameters of bipolar transistors 2N2222. The prediction uses a general database of existing radiation tests whose samples belong to different assembly lots. Machine learning methods allow for determining the several degradation patterns that are present in the database. After the recognition of existing patterns, the only input to predict the degradation of pristine samples is to measure electrical parameters in the absence of radiation. The latter largely simplifies the experimental set-up to perform radiation testing, reduces the cost of the process, and does not invalidate the samples for later use. The objective of this article is to present the work as an example of the procedure. To simplify the problem, a generic component with multiple uses in space projects, the 2N2222, has been used. However, the methodology can be applied to any other device at any time. The studies published to date, along with the work developed this far, has enabled the modelling of the degradation behaviour of different parameters for different devices, thereby adding new patterns to the model-based approach. For instance, a new pattern was introduced for modelling the non-monotonic behaviour observed in operational amplifier parameters. As a result, we trust the reliability of this methodology, even if minor modifications are required to add new patterns to APEP to cluster the data. It is important to highlight that APEP is a non-destructive technique, so its application would not reduce the number of available flight or qualification models under any circumstances.

It should be noted that the identification of a large enough number of degradation patterns requires the use of a large database of measurements (the larger the better). Therefore, an essential objective to promote the use of COTS devices as cheaper alternatives to RHA devices is the development of large radiation tests databases like, for example, the PRECEDER database.

APEP has been applied to samples under two types of irradiation conditions: biased and unbiased. On the other hand, two different approaches have been tested: pure data-driven and model-based. Table 6 and Table 7 summarise the good and comparable results obtained in the several combinations between irradiation conditions and approaches. Therefore, it is possible to conclude that APEP is a successful method to predict the degradation of new samples. In the tests that have been carried out, the clustering process has been shown to be very efficient to recognise the several degradation patterns that are present in the training samples. This is especially important in the case of the data-driven approach, whose main issue is the scarcity of training samples.

When the sample degradation can be fitted to particular models, the heterogeneity of the databases (in relation to the accumulated dose) is not so critical. This can be seen by simple inspection of Table 3 and Table 5. Data-driven approaches require that all samples reach the same dose values and, in general, it causes a severe restriction in the number of useful samples to train the unsupervised classifier. However, if the degradation follows simple models (i.e., with few parameters), the fit does not require the use of the same dose values and the number of available samples for the clustering process significantly increases.

It should be noted that the example of Figure 10 (model-based approach) can be compared to the example of Figure 4b (data-driven approach) as they represent the same samples. Firstly, it is important to mention that the average degradation patterns (black continuous lines) are equivalent. Secondly, it is necessary to underline that the error band is narrower for the same confidence level in the case of the model-based approach. This means a lesser uncertainty in the prediction of parameters at any dose. Thirdly, an additional comparison between model-based and data-driven approaches can be raised. To know the electrical parameter value at a specific dose, data-driven approaches require the use of linear interpolation. However, when a model is available, its parameters are known and, therefore, no interpolation is required.

All the examples in this study have been compatible with an exponential degradation model. But of course, APEP can be applied with any other models that fit the data behaviour. Also, the good results of APEP allow its application even in cases where the data cannot be fitted to any model. In these cases, the data-driven approach can be used.

As a corollary, the APEP method has demonstrated sufficient capacity to develop a methodology to predict the degradation of any microelectronic component, even though in this work, only a specific type has been shown. In any case, it is important to emphasise that for a successful recognition of degradation patterns, it is essential to increase as much as possible data repositories such as the PRECEDER database.

## Figures and Tables

**Table 1:** Example of electrical quantities to be measured in the case of a bipolar transistor of type 2N2222 and limits specified by the manufacturer (Microsemi, Aliso Viejo, CA, USA).

Parameter	Symbol	Conditions	Min Limit	Max Limit	Unit
Collector to Base cutoff current	ICBO_1_	VCB = 75 V		10,000	nA
	ICBO_2_	VCB = 60 V		10	
Emitter to base cutoff current	IEBO_1_	VEB = 6 V		10,000	
	IEBO_2_	VEB = 4 V		10	
Collector to emitter cutoff current	I_CES_	VCE = 50 V		50	
Forward-current transfer ratio	hFE_1_	VCE = 10 V, IC = 0.1 mA	50		
	hFE_2_	VCE = 10 V, IC = 1 mA	75	325	
	hFE_3_	VCE = 10 V, IC = 10 mA	100		
	hFE_4_	VCE = 10 V, IC = 150 mA	100	300	
Collector-Emitter saturation Voltage	V_CE(SAT)_	IC = 150 mA, IB = 150 mA		300	mV
Base-Emitter Saturation Voltage	V_BE(SAT)_		600	1200	mV

**Table 2:** Degradation models: parameter amplitude as a function of the irradiation dose $\left(x\right)$.

Model	Equation	Coefficients
Linear	$a_{1}+a_{2}\cdot x$	$a_{1}:$ amplitude at 0 krad $a_{2}:$ slope
Exponential	$a_{2}+\left(a_{1}-a_{2}\right)\exp\left(-a_{3}\cdot x\right)$	$a_{1}:$ amplitude at 0 krad $a_{2}:$ asymptotic value at x>> $a_{3}:$ rate of change
Sigmoid	$a_{4}+\frac{a_{2}-a_{4}}{1+\left(\frac{a_{2}-a_{4}}{a_{1}-a_{4}}-1\right)\exp\left(-a_{3}\cdot x\right)}$	$a_{1}:$ amplitude at 0 krad $a_{2}:$ asymptotic value at x>> $a_{3}:$ rate of change $a_{4}:$ asymptotic value at x<<

**Table 3:** Available records for each electrical parameter and irradiation condition.

Electrical Parameter	Irradiation Conditions	Database Records
hFE_1_	Biased	223
	Unbiased	131
hFE_2_	Biased	218
	Unbiased	126
hFE_3_	Biased	223
	Unbiased	131
hFE_4_	Biased	223
	Unbiased	131

**Table 4:** Samples inside the same cluster show the highest similarity.

Cluster Colour	Average Intra-Cluster Similarity	Average Inter-Cluster Similarity
Orange	0.99991	0.90338
Blue	0.99953	0.89751
Cyan	0.99913	0.95647
Green	0.99414	0.90146
Red	0.99172	0.93038

**Table 5:** Available records for training/test purposes for two approaches: data-driven and model-based.

Electrical Parameter	Irradiation Conditions	Database Records	Largest Set of Records for 6 Common Irradiation Doses	Remaining Set of Records for an Exponential Degradation Model
hFE_1_	Biased	216	65 (29.1%)	172 (77.1%)
	Unbiased	121	65 (49.6%)	111 (84.7%)
hFE_2_	Biased	213	65 (29.8%)	167 (76.6%)
	Unbiased	116	65 (51.6%)	104 (82.6%)
hFE_3_	Biased	218	65 (29.1%)	160 (71.7%)
	Unbiased	121	65 (49.6%)	90 (68.7%)
hFE_4_	Biased	218	65 (29.1%)	133 (59.6%)
	Unbiased	121	65 (49.6%)	90 (68.7%)

**Table 6:** Success rates obtained with 100 different datasets in a data-driven approach. Each dataset is made up of 30% of the available data (the remaining 70% has been used for training).

Electrical Parameter	Irradiation Conditions	Min	Median	Mean	Max
hFE_1_	Biased	46.49	66.67	67.92	87.72
	Unbiased	43.86	64.04	64.61	88.60
hFE_2_	Biased	59.65	76.32	76.25	88.60
	Unbiased	44.74	63.16	63.01	80.70
hFE_3_	Biased	57.89	82.46	82.44	96.49
	Unbiased	43.86	71.93	71.28	90.35
hFE_4_	Biased	70.18	90.35	88.81	100.00
	Unbiased	57.02	80.70	79.09	92.98

**Table 7:** Success rates obtained with 100 different datasets in a model-based approach. Each dataset is made up of 30% of the available data (the remaining 70% has been used for training).

Electrical Parameter	Irradiation Conditions	Min	Median	Mean	Max
hFE_1_	Biased	62.14	73.04	72.70	83.64
	Unbiased	71.79	84.01	84.40	95.61
hFE_2_	Biased	66.67	80.25	79.58	89.84
	Unbiased	60.85	81.02	80.19	91.11
hFE_3_	Biased	76.36	91.43	90.62	96.67
	Unbiased	66.67	88.53	87.54	96.32
hFE_4_	Biased	75.45	89.06	88.12	97.78
	Unbiased	78.86	93.68	92.89	100.00

## Data Availability

Data are contained within the article.
